# A new neurosurgical adjustable pressure suction apparatus with a mechanically controlled air inlet: the study of precise regulation and atraumatic suction

**DOI:** 10.3389/fneur.2022.979494

**Published:** 2022-09-20

**Authors:** Pan Nie, Mengxi Chen, Jibo Zhang, Meicheng Pan, Xiqian Gu, Chengshi Xu, Zhengwei Li, Jianjian Zhang, Wenyuan Zhao, Xiang Li, Jie Zhang, Jincao Chen

**Affiliations:** ^1^Department of Neurosurgery, Zhongnan Hospital of Wuhan University, Wuhan, China; ^2^Brain Research Center, Zhongnan Hospital of Wuhan University, Wuhan, China; ^3^Department of Radiology, Zhongnan Hospital of Wuhan University, Wuhan, China; ^4^Department of Anesthesiology, Zhongnan Hospital of Wuhan University, Wuhan, China; ^5^Frontier Science Center for Immunology and Metabolism, School of Medicine, Medical Research Institute, Wuhan University, Wuhan, China

**Keywords:** suction, suction tube, microsurgery, neurosurgery, oncology

## Abstract

**Background:**

An essential surgical tool in neurosurgery is the suction tube. The skillful and accurate use of a suction tube facilitates the neurosurgical operation.

**Objective:**

This study is to verify the practicality of an adjustable pressure suction tube (APS tube) and to explore the ideal APS tube diameter and tip negative pressure for different intracranial structures.

**Methods:**

APS tubes were used to aspirate brain tissues and carotid arteries of rats. Laser speckle contrast imaging (LSCI) was used to record the blood flow velocity (BFV). We measured APS tube diameter, air inlet size, tip negative pressure and central negative pressure and calculated the correlation between them. In our department, intraoperative real-time parameters including APS tube diameter, length, air inlet size, and central negative pressure were recorded, and the tube tip negative pressure suitable for different intracranial structures and parts was calculated.

**Results:**

All experiments were carried out using APS tubes. Experiments on rats objectively reflected a severe structural damage to the brain and blood vessels by the suction tube, which might even result in an irreversible reduction in blood flow., Rat carotid arteries and brain tissue suffered severe damage when the tip negative pressure exceeded 33.4 ± 1.8 and 29.2 ± 2.0 kPa, respectively. BFV failed to return to the preoperative level 3 min after the operation (*p* < 0.05), and this decrease was more pronounced when the suction tube diameter was large (*p* < 0.05). The tip negative pressure was positively and negatively correlated with central negative pressure and the air inlet size, and was independent of APS tube diameter. A total of 50 operations including 39 tumor resection operations and 11 moyamoya disease bypass operations were recorded. Large-diameter APS tubes (3.5 mm) with an closed air inlet were frequently used to maintain a greater tip negative pressure before the incision of dura mater. When important structures such as motor cortex and brainstem were involved, 1.5- or 2.0-mm-diameter APS tubes were mostly used, and an air inlet was opened up to 0.7–2.1 mm to maintain a safe tip negative pressure (7.4–27.9 kPa).

**Conclusion:**

APS tubes with a mechanical knob provide stable and precise adjustment of the tip negative pressure, avoiding excessive negative pressure that causes serious damage to the intracranial structure. And, this allows the surgeon to hold the suction tube more freely and operate at any angle with an appropriate fulcrum near the incision to achieve efficient atraumatic suction and enhance surgical safety.

## Background

**Figure 1:**
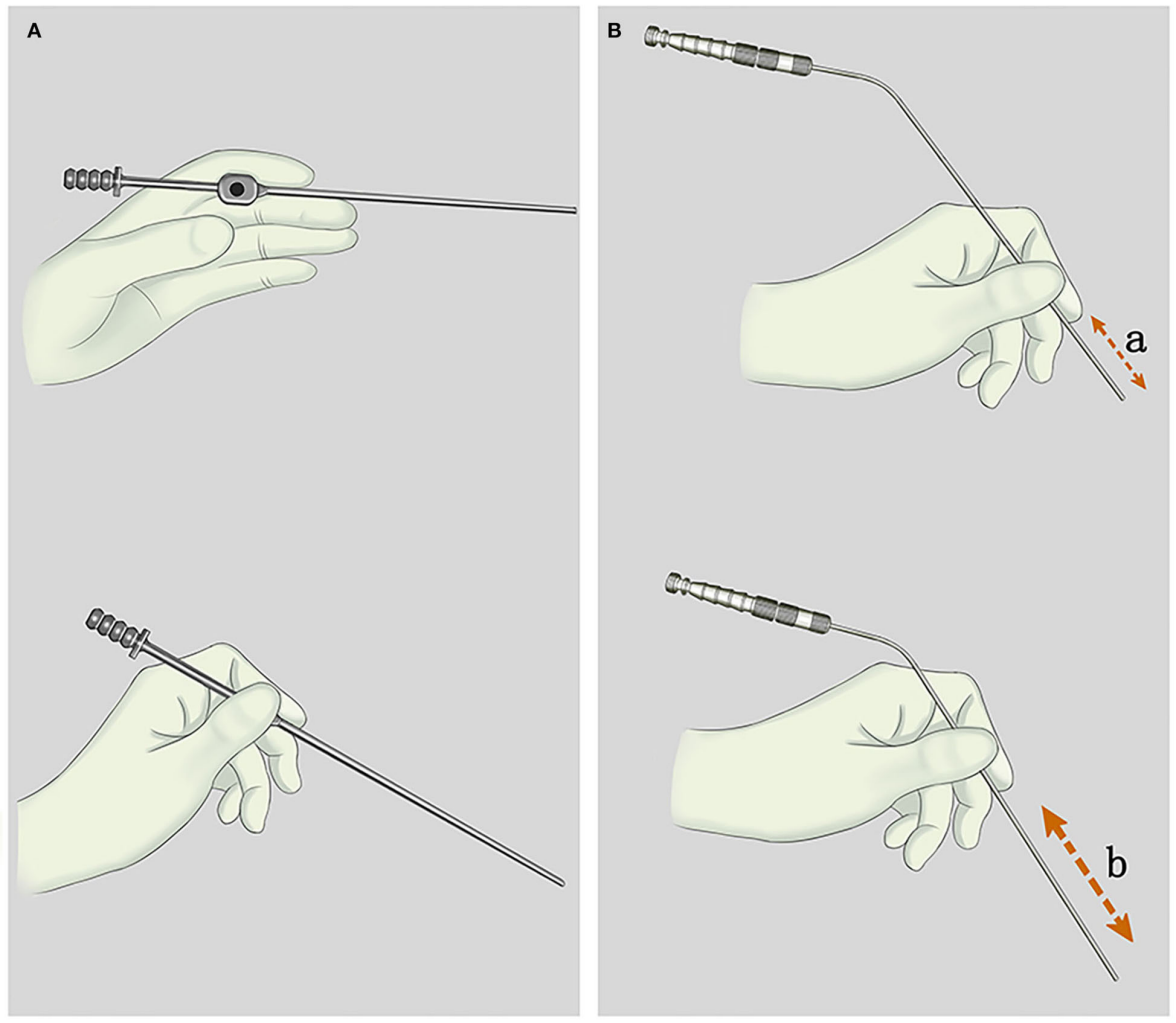
**(A)** The style of holding the traditional suction tube. When the thumb is placed on the air inlet, the actual working distance of the suction tube is extended, which reduced the stability and accuracy of operation. **(B)** The style of holding an adjustable pressure suction tube (APS tube). “a and b” represent the actual working length of APS tubes, which is adjustable.

Neurosurgical operation is characterized by a small, narrow, and deep surgical field with complex anatomy (1). Since Dr. Theodore Kurze performed the first acoustic neuroma surgery under a microscope in 1957, microsurgery has gradually evolved into another name for neurosurgery (2). The suction tube, an indispensable operative instrument in neurosurgery, helps to keep the surgical field clean by suctioning out liquid, blood, and tissue fragments, and also plays a role in pulling and removing lesions (3). The suction tube is usually provided with an air inlet, traditionally controlled by the thumb, to adjust the tube tip negative pressure, which is proven to be unpredictable and variable and reduced suction capacity and efficiency by obstructing flow (4). What is worse, it limits the way surgeons hold the suction tube. When the thumb is placed on the air inlet, the actual working distance of the suction tube is extended, which reduces the stability and accuracy of the operation (Figure 1A).

Although a study reported that the threshold value for a safe negative pressure of dog brain tissue was approximately 15 kPa, it could not represent the ideal negative pressure for different structures of the human brain (5). On the other hand, the negative pressure at the suction tip cannot be predictable and is variable, depending on the air inlet and the load on the central vacuum system. The application of an intraoperative suction tube, including the adjustment of tube length, diameter, and tip negative pressure, has always been based on the experience of the surgeon.

Therefore, our department developed a new suction tube named an adjustable pressure suction tube (APS tube) with a mechanically controlled air inlet. Additionally, we explored the ideal APS tube diameter, air inlet size, and tip negative pressure for different intracranial structures and parts.

## Materials and methods

### APS tubes

**Figure 2:**
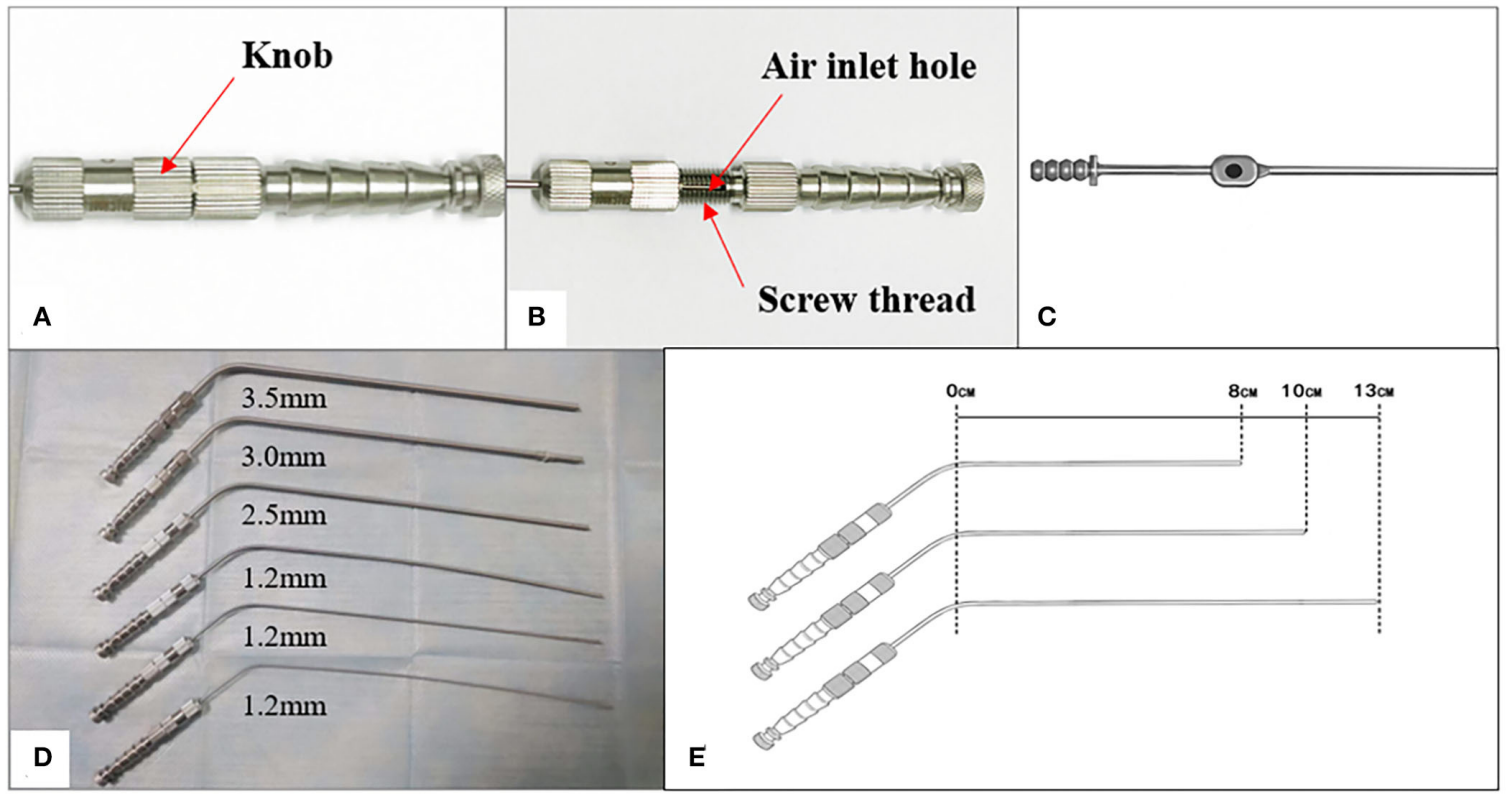
**(A,B)** An APS tube's air inlet is controlled by a mechanical knob. **(C)** An air inlet of the traditional suction tube. **(D,E)** The diameter and working length of an APS tube.

Unlike conventional suction tubes, the air inlet in APS tubes is controlled by a mechanical knob with the ability to quantify the hole size and maintain a constant value (Figures 2A–C). The rectangular air inlet measures 1 mm in width and 6.5 mm in length and corresponds to nine screw threads. These tubes are manufactured with outer and inner diameters in dimensions of 1.2–3.5/0.8–3.1 mm and working lengths of 8–13 cm (Figures 2D,E, Table 1).

**Table 1:** Diameters of APS-tube.

**Inner**	**Outer**
0.8 mm	1.2 mm
1.1 mm	1.5 mm
1.6 mm	2.0 mm
2.1 mm	2.5 mm
2.6 mm	3.0 mm
3.1 mm	3.5 mm

### Animals

Protocols for the animal experiments were reviewed and approved by the Institutional Animal Care and Use Committee. We set up an independent negative pressure system consisting of an electric air pump (0–80 kPa), a digital manometer, an APS tube, and connecting catheters.

#### Experimental procedures

**Figure 3:**
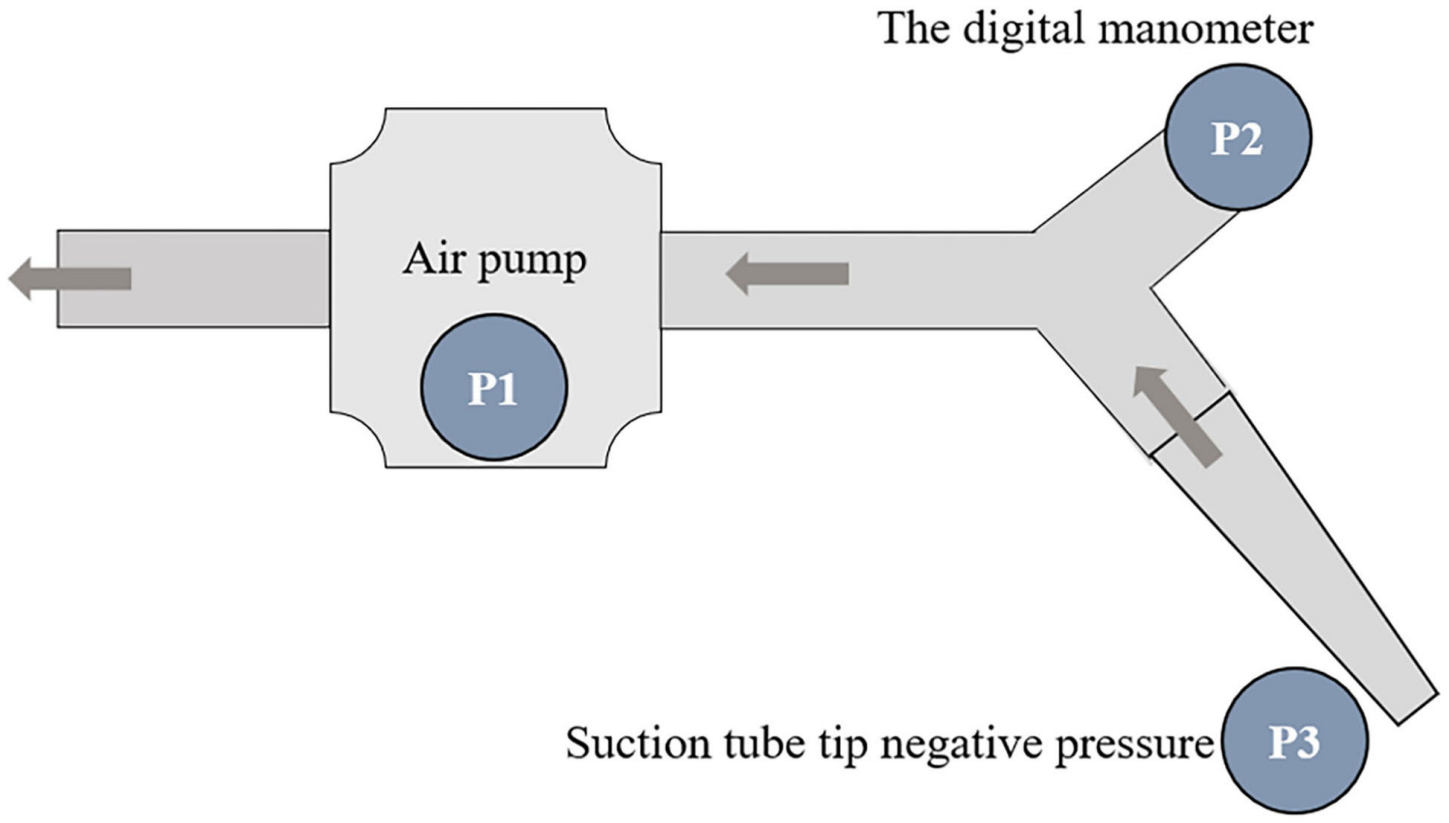
The working mode diagram of an independent negative pressure system. P2 was measured by a digital manometer. P3 stood for the tip negative pressure, which was equal to P1 and P2, when the negative pressure system became vacuum.

The negative pressure system is connected (Figure 3).

Rats were anesthetized with 10% chloral hydrate intraperitoneal infiltration (4 ml/kg) and fixed.

The carotid artery of rats was dissected under a microscope, and a small piece of rubber glove was placed under the carotid artery to facilitate observation and operation. Carotid blood flow velocity (BFV) was recorded by laser speckle contrast imaging (LSCI). LSCI is based on the blurring of interference patterns of the scattered laser light by the flow of blood cells to instantly visualize blood perfusion in the microcirculation (6).

We measured the diameter of the rat carotid artery and selected a suction tube (1.2 mm diameter) to match the measured diameter. An air inlet of the suction tube was closed during this experiment. When we turn on an electric air pump and block a tip of the suction tube, an independent negative pressure system will gradually become vacuum, which means that pressures in each part of the system are equal and can be determined by the equal values of a digital manometer and an air pump. The value at this point is the maximum tip negative pressure for a single adjustment. Initially, an air pump and suction tip negative pressure were adjusted to a minimum. The tube tip was opened and held it firmly against the carotid arterial wall for 30 s until it was gently separated. Changes in the carotid artery morphology and BFV were recorded by laser speckle. And then, the negative pressure was gradually increased, and the abovementioned steps were repeated until the carotid artery deformation was so severe that the bulge on the carotid artery wall could not be recovered after the suction tube was removed. We recorded the BFV again for 3 min after the operation.

The suction tube was replaced with a diameter of 2.0 -mm (an inner diameter of 1.6 mm) and step 4 was repeated.

Rats were executed by cervical dislocation. We dissected the intact brain and isolated the pia mater. The operation of a suction tube in step 4 was repeated, and the morphological changes in the brain tissue were observed. A 2.0-mm-diameter suction tube (an inner diameter of 1.6 mm) was used.

All rat surgeries in the whole study were performed by the same skilled researcher.

### Measurement of APS tube parameters

Variables related to suction tube tip negative pressure included central negative pressure and suction tube diameter and air inlet size. We measured and calculated the relationship between suction tube tip negative pressure and each variable separately using the control variable method.

### Recording of intraoperative real-time data

We recorded suction tube diameter, air inlet size, and central negative pressure in the scalp, skull, dura mater, cerebellopontine angle (CPA) region, cavernous sinus, brainstem, and in lesions during different surgeries. The air inlet size is expressed by the number of threads (one screw thread = 0.7 mm). All surgeries were performed by a very experienced surgeon. We estimated intraoperative real-time tip negative pressure using the data model obtained from previous experiments. We also recorded the video and corresponding electrophysiological changes when the suction tube tip was operated in special parts such as brainstem and motor cortex.

### Statistics

SPSS 22.0 was used for statistical analysis of the data. Changes in BFV were analyzed using the paired sample *t*-test. Data were expressed as mean ± standard deviation (SD). Values of *p* < 0.05 were considered significant.

## Results

### Assessment of injury degree of the carotid artery and brain tissue in rats under different negative pressures

**Figure 4:**
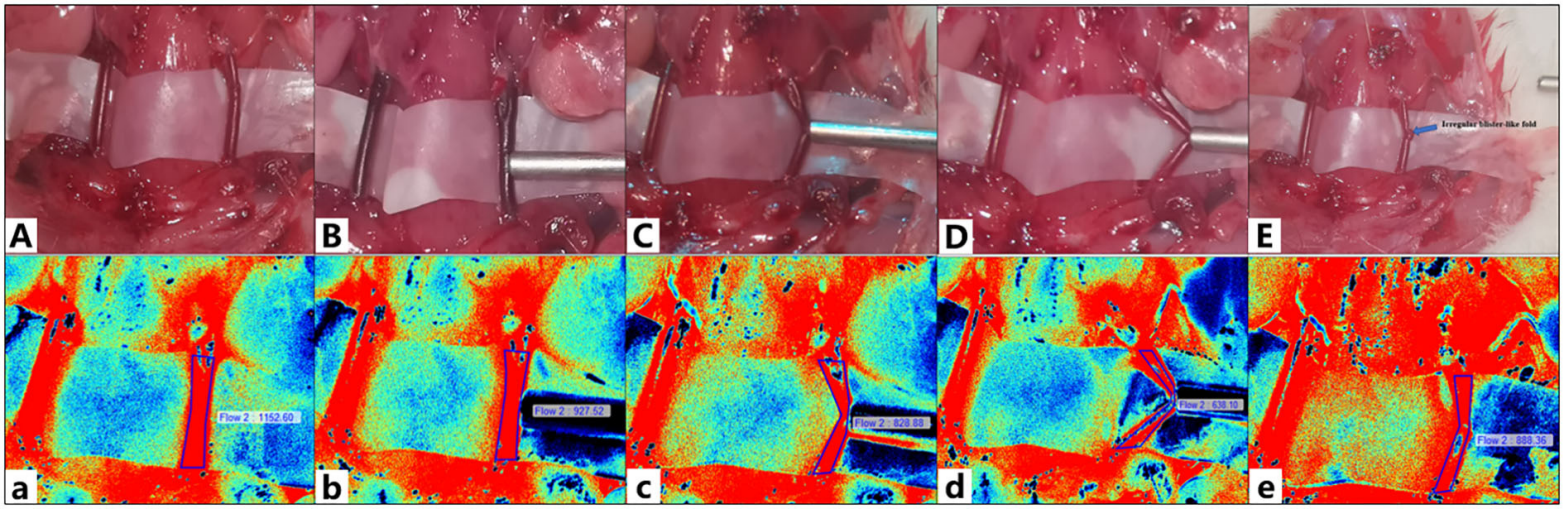
The camera captured **(A–E)**. Laser speckle contrast imaging (LSCI) was used to capture **(a–e)**. **(A,a)** The carotid artery and its blood flow before suction. **(B,b)** Type A deformation of blood vessels. **(C,c)** Type B deformation of blood vessels. **(D,d)** Type C deformation of blood vessels. **(E,e)** The carotid artery and its blood flow after suction. The blue arrow indicates an irregular blister-like fold on the vessel wall.

The diameter of the rat carotid artery was approximately 1.08 ± 0.11 mm (Table 2). We classified the deformation of blood vessels caused by suction tubes into types A, B, and C (Figure 4). There was no morphological change in Type A vessels, and the tip negative pressure was <17.4 ± 2.2 kPa; type C vessels were severely bent and an irregular blister-like bulge appeared after removing the suction tube, which could not be completely recovered, and the tip negative pressure was > 33.4 ± 1.8 kPa; type B vessels were slightly curved, and there was no obvious bulge on the vessel wall, when the tip negative pressure was 17.4–33.4 kPa. The BFV before the procedure was 1,032.07 ± 61.37. When a 1.2-mm-diameter suction tube was adopted, type A BFV was 969.53 ± 68.16 (*p* < 0.05); type B BFV was 849.62 ± 60.01 (*p* < 0.05); type C BFV was 660.59 ± 51.79 (*p* < 0.05), and the BFV was 861.96 ± 47.12 for 3 min after the suction tube was removed (*p* < 0.05). In addition, when the diameter of a suction tube was changed to 2.0 mm, there was a more significant decrease in the blood flow (*p* < 0.05, Table 3).

**Table 2:** Diameter of rat's carotid artery.

**Rats**	**Weight (g)**	**Carotid artery diameter (mm)**
1	180	1.12
2	200	1.19
3	200	1.07
4	200	0.98
5	180	0.91
6	200	1.20
Mean	193	1.08 ± 0.11

**Table 3:** Blood flow of rat's carotid arteries.

**Suction tube diameter**	**Type**	**Blood flow**	*P*–value^*^	Blood flow reduction^**^	*P*–value^***^
1.2 mm	Before procedure	1032.07 ± 61.37			
	A	969.53 ± 68.16	0.001	62.54 ± 20.66	
	B	849.62 ± 60.01	0.000	182.45 ± 27.57	
	C	660.59 ± 51.79	0.000	371.48 ± 43.64	
	3 min after procedure	861.96 ± 47.12	0.000	170.11 ± 21.71	
2.0 mm	Before procedure	1043.37 ± 66.61			
	A	837.39 ± 46.87	0.000	205.99 ± 28.36	0.000
	B	715.77 ± 58.39	0.000	327.60 ± 27.73	0.000
	C	536.86 ± 52.78	0.000	506.51 ± 29.03	0.001
	3 min after procedure	785.56 ± 67.51	0.000	257.81 ± 25.65	0.003

* Comparison of blood flow after and before procedure.

** Reduction of blood flow before and after procedure.

*** Comparison of blood flow reduction after suction with different diameter of APS-tubes (1.2 vs. 2.0 mm).

**Figure 5:**
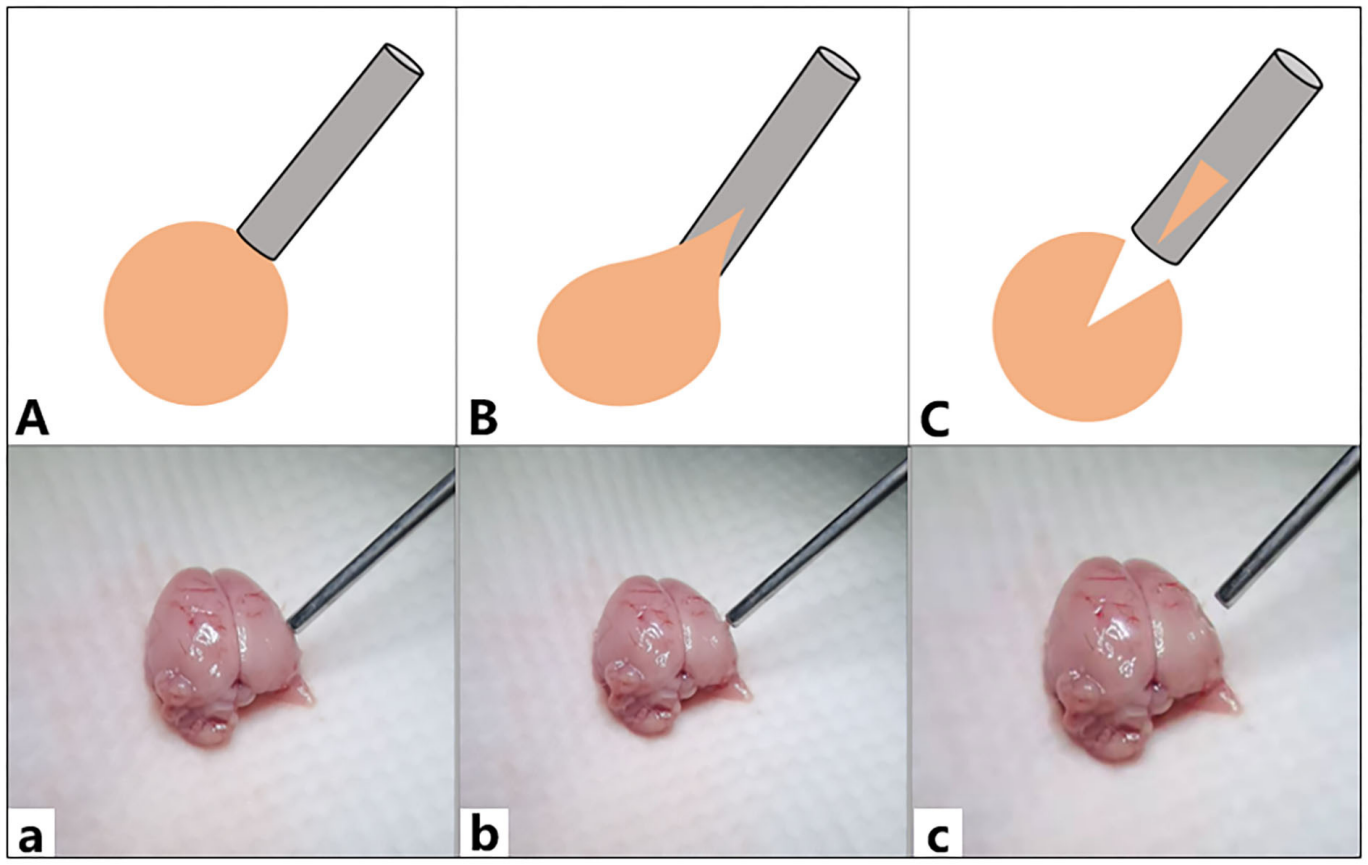
Graph showing the damage of rat brain tissue. **(A,a)** The tip negative pressure was <5.2 ± 0.7 kPa, and there was no significant damage to the brain tissue. **(B,b)** The tip negative pressure was 5.2–29.2 kPa, and a mild destruction on the brain tissues was observed. **(C,c)** The tip negative pressure was > 29.2 ± 2.0 kPa, and the suction tube could directly suction away the brain tissue causing a severe damage.

Compared with the carotid artery, the brain tissue of rats was more fragile. When the tip negative pressure was <5.2 ± 0.7 kPa, there was no significant damage to the brain tissue. When the tip negative pressure was 5.2–29.2 kPa, mild brain tissue destruction was observed. When the tip negative pressure was > 29.2 ± 2.0 kPa, the suction tube could be used to directly suction out the brain tissue and cause a significant damage (Figure 5).

### Assessment of the APS tube parameters

**Figure 6:**
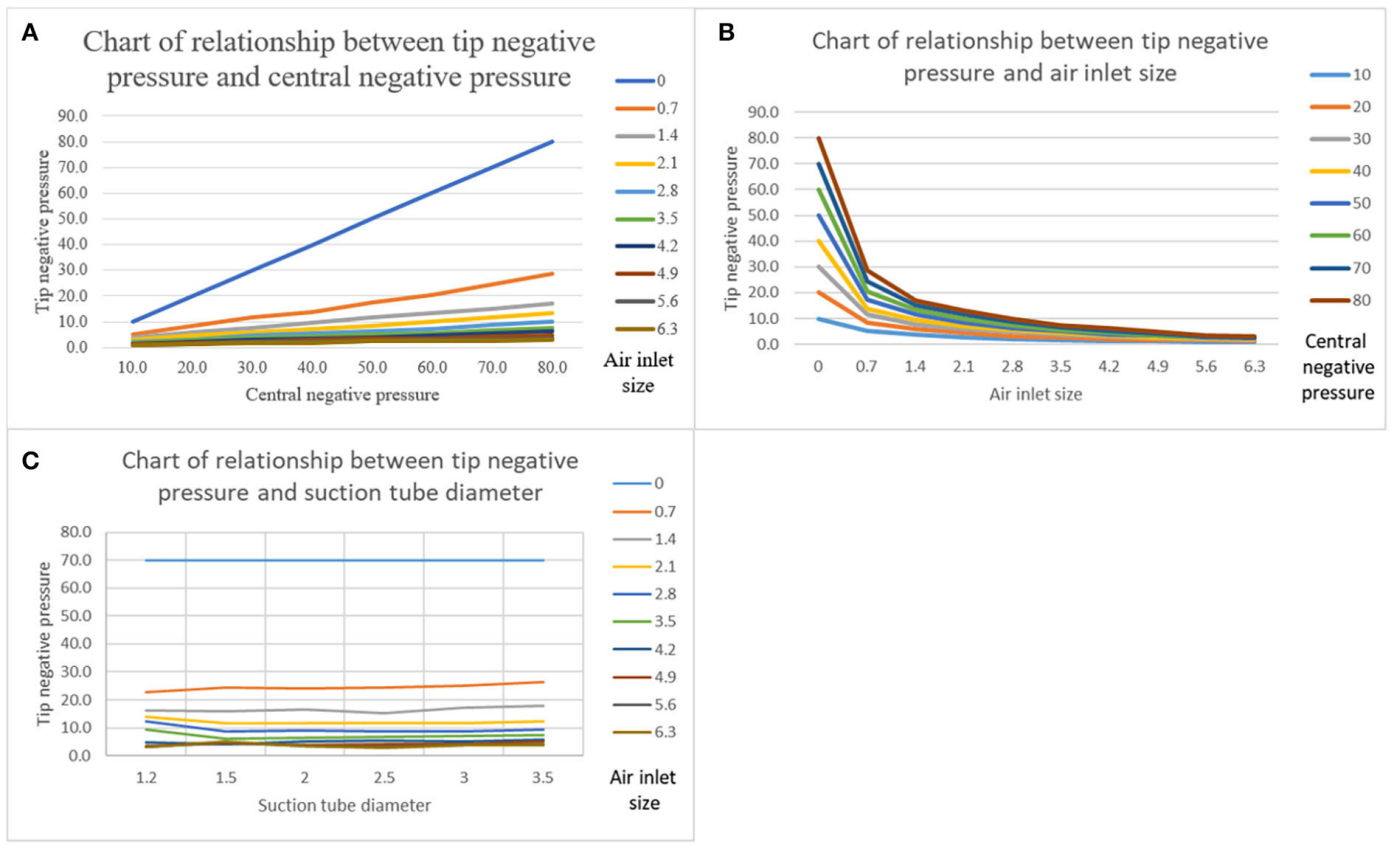
**(A)** A chart showing the relationship between the tip negative pressure and central negative pressure. Suction tube diameter was 2.5 mm. **(B)** A chart showing the relationship between the tip negative pressure and the air inlet size. Suction tube diameter was 2.5 mm. **(C)** Chart showing the relationship between the tip negative pressure and suction tube diameter. Central negative pressure was 70 kPa.

Adjustable pressure suction tube tip negative pressure was positively and negatively correlated with central negative pressure (Figure 6A), and the air inlet size (Figure 6B), and was independent of suction tube diameter (Figure 6C). The tip negative pressure is almost proportional to the central negative pressure and is shown in a line chart. Additionally, when an air inlet was sufficiently large, it would have little effect on the tip negative pressure.

### Intraoperative real-time recording of APS tubes

A total of 50 operations including 39 tumor resection operations and 11 superficial temporal artery to middle cerebral artery (STA-MCA) bypass operations for moyamoya disease were recorded. Central negative pressure was maintained between 40 and 80 kPa in our operating room. The working length of APS tubes in all operations was 13 cm. For operating on the scalp, skull, and epidural space, a 3.5-mm-diameter APS tube was used, and its air inlet was completely closed. The maximum tip negative pressure was 40 to −80 kPa. When operating on the lateral fissure, a 2.0 or 2.5-mm-diameter APS tube was mostly used, and its air inlet was completely closed. The maximum tip negative pressure was 40–80 kPa. In 11 cases of STA-MCA bypass, a 1.5- or 2.0-mm-diameter APS tube with a diameter of was mostly adopted. The air inlet size was adjusted to 2.1–5.6 mm (three to eight screw threads), and the maximum tip negative pressure was 3.1–13.0 kPa. When operating on the junction area between the tumor and motor/sensory cortex, a 2.0-mm-diameter APS tube with the air inlet size adjusted to 0.7–1.4 mm (one to two screw threads) was used, and the maximum tip negative pressure was 9.7–27.9 kPa. When operating on the junction area between the tumor and brain stem, a 1.5- or 2.0-mm-diameter APS tube was mostly adopted. The air inlet size was adjusted to 0.7–1.4 mm (one to two screw threads), and the maximum tip negative pressure was 9.7–27.9 kPa. When the cavernous sinus and CPA regions were involved in the operation, the 1.5- or 2.0-mm-diameter APS tube was mostly used. The air inlet size was set as 0.7–2.1 mm (one to three screw threads), and the maximum tip negative pressure was 7.4–27.9 kPa. However, when occurring inside the tumor, 2.0- or 2.5-mm-diameter APS tubes with a completely closed air inlet were mostly used, and the maximum tip negative pressure was 40–80 kPa (Table 4).

**Table 4:** Intraoperative real-time recording of APS-tube.

**Position**	**Diameter (mm)**	**Number of threads**	**Air inlet size (mm)**	**maximum tip negative pressure (kpa)**	**Length (cm)**	**Number**
Epidural: scalp, skull, epidural space	3.5	0	0	40.0–80.0	13	50
Lateral fissure	2.0/2.5	0	0	40.0–80.0	13	17
Moyamoya disease						11
STA–MCA bypass	2.0	3–6	2.1–4.2	3.6–13.0	13	4
	1.5	5–8	3.5–5.6	3.1–7.0	13	7
Tumor						39
Inside the tumor	2.0	0	0	40.0–80.0	13	24
	2.5	0	0	40.0–80.0	13	15
Tumor–Motor/Sensory cortex	2.0	1–2	0.7–1.4	9.7–27.9	13	8
Tumor–Brainstem	1.5	1	0.7	13.7–27.6	13	4
	2.0	1–2	0.7–1.4	9.7–27.9	13	3
Tumor–Cavernous sinus/CPA	1.5	1–2	0.7–1.4	9.2–27.6	13	9
	2.0	1–3	0.7–2.1	7.4–27.9	13	9

STA, Superficial temporal artery; MCA, Middle cerebral artery; CPA, Cerebellopontine angle.

### Case report

**Figure 7:**
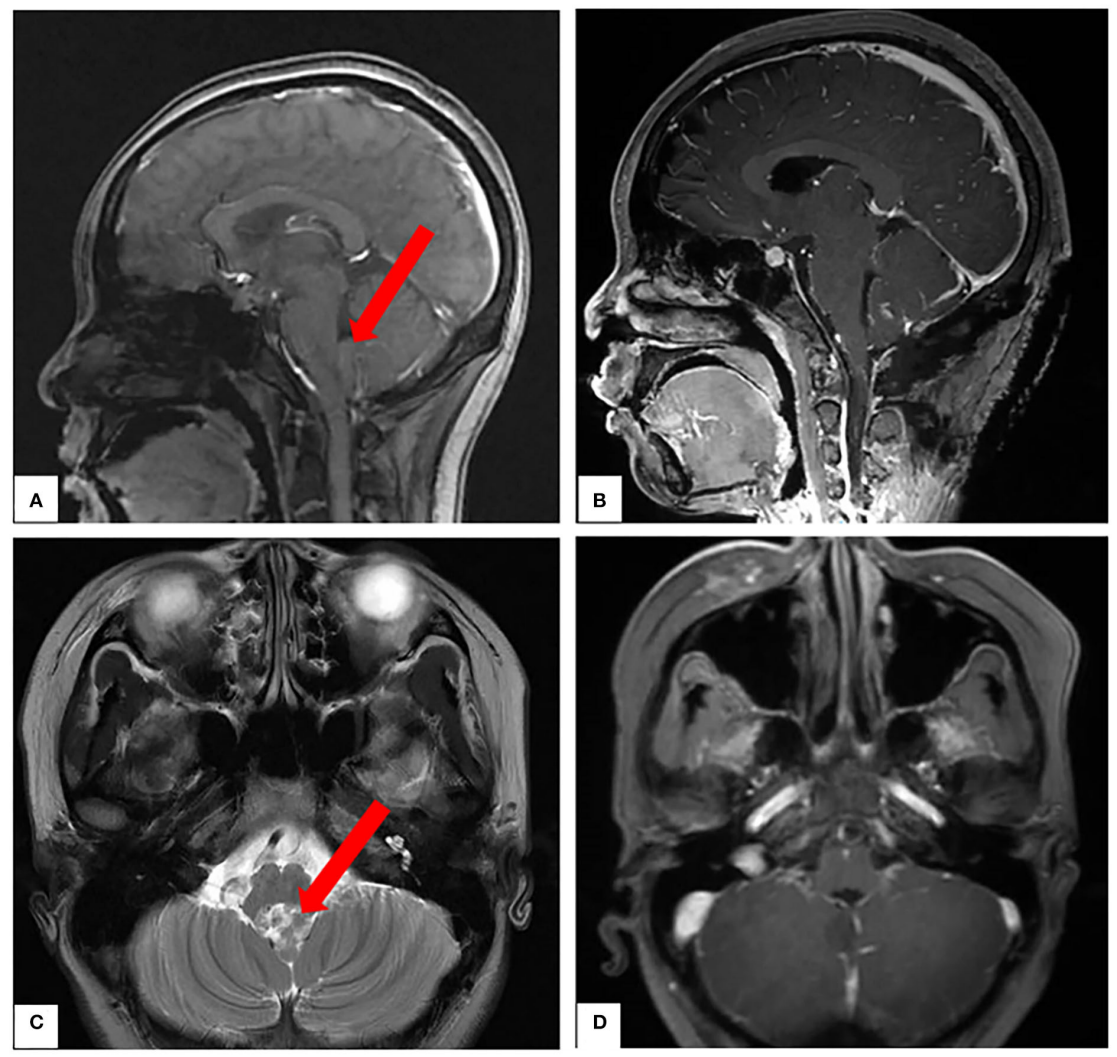
**(A,C)** Preoperative magnetic resonance imaging (MRI). **(B,D)** Postoperative MRI.

**Figure 8:**
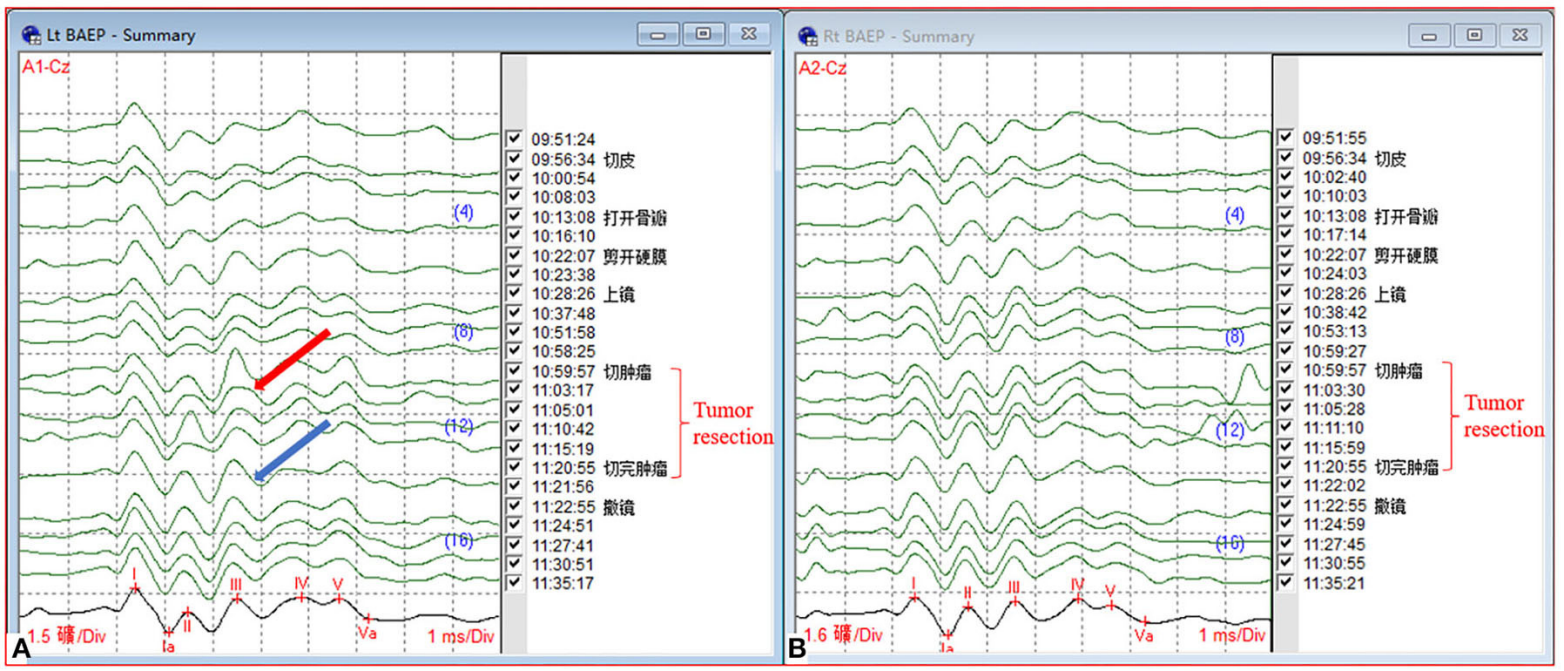
Graph of intraoperative electrophysiological records of brainstem auditory-evoked potentials (BAEP). **(A)** Left BAEP. Red arrow: at the beginning of a tumor resection, the amplitude decreases; Blue Arrow: at the end of a tumor resection, the amplitude recovers. **(B)** Right BAEP.

A patient in her mid-teens was found to have a space-occupying lesion in the fourth ventricle on physical examination, abutting, and squeezing the brainstem (Figure 7). She recovered well after a surgical resection in our department. According to the pathology report, it was an atypical choroid plexus papilloma. During a tumor resection, the patient's left brainstem auditory-evoked potentials (BAEP) decreased and recovered (Figure 8), which reflected that the APS tube could be used to suction out and remove the tumor without damaging the brainstem by maintaining the ideal tip negative pressure (Supplementary Video 1).

## Discussion

### Inappropriate use of suction tubes causes damage to the brain and blood vessels

It is unethical to obtain the ideal negative pressure range of normal intracranial structures through in *vivo* human experiments. Also, the negative pressure range of the suction tube tip obtained from the experiment on rats is not suitable for humans. However, it objectively reflected the severe structural damage to the brain and blood vessels by the suction tube, which may even result in an irreversible reduction in BFV. Moreover, the damage would be more severe if the suction tube diameter was large. In addition to the physical damage caused by the suction tube, the vasospasm caused by such damages can also cause the irrecoverable blood flow. We should reduce the incidence of this situation, especially in patients with a high risk of vasospasm, such as subarachnoid hemorrhage, which can be fatal (7).

### Characteristics of the APS tube

**Figure 9:**
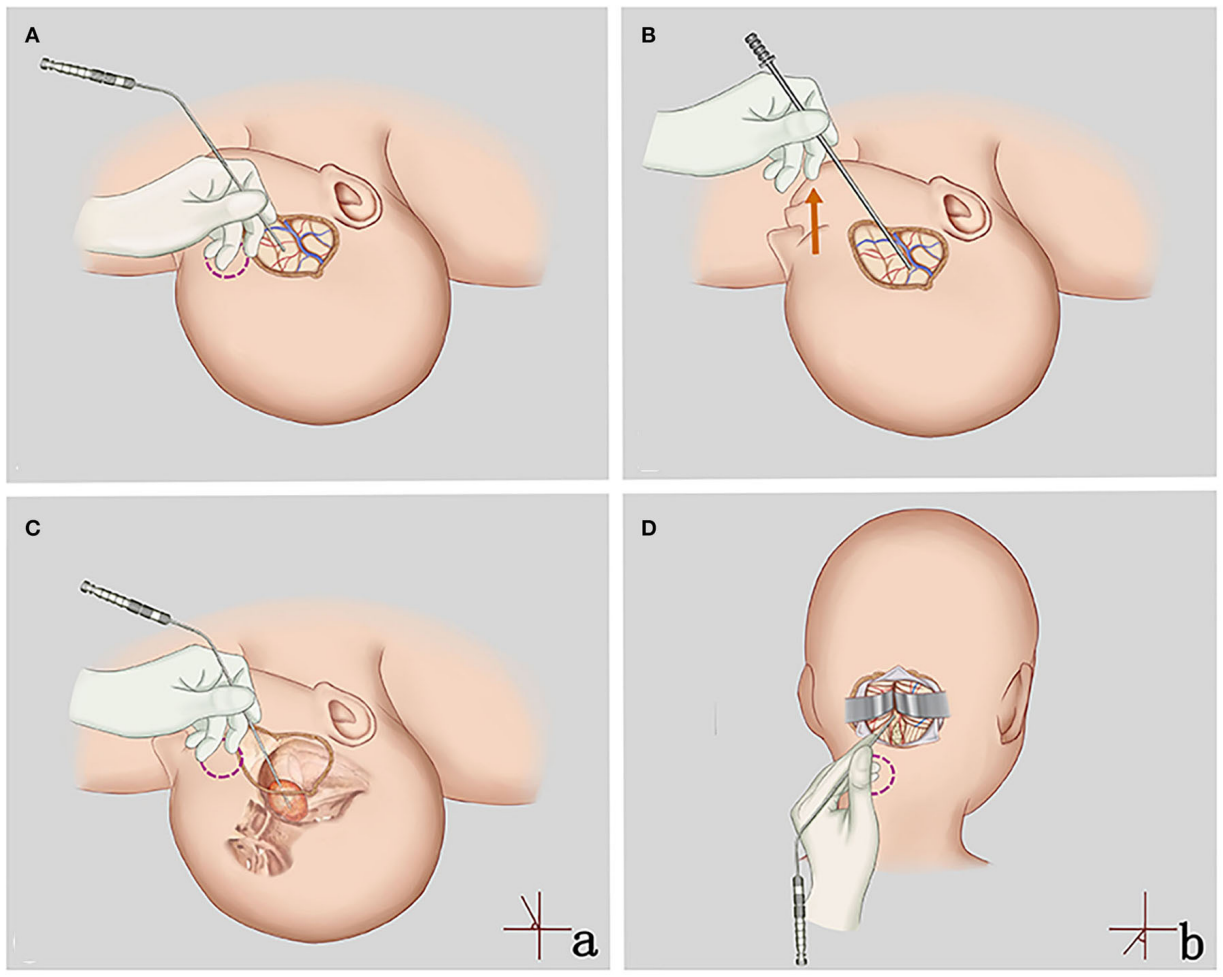
**(A,C,D)** Application of the APS tube for superficial and deep craniotomy. Neurosurgeons can flexibly adjust the angle **(a,b)** and the actual working length of the APS tube and find an appropriate fulcrum (red circles), which is conducive to safe, stable, and accurate surgery. **(B)** Application of the traditional tube for superficial craniotomy. The surgeon's hand is suspended and unstable (red arrow).

The air inlet is to facilitate the surgeon to adjust the tube tip negative pressure. APS tubes with a mechanical knob instead of the thumb to control the air inlet size has two advantages: (1) The air inlet size can be continuously and stably adjusted and kept constant, and the tube tip negative pressure is more predictable and controllable and (2) the surgeon can hold the suction tube more freely as the thumb is liberated, which shortens the actual working distance of the suction tube and allows the surgeon to operate at any angle with an appropriate fulcrum near the incision (Figure 9). Therefore, this new suction tube achieves stable and precise adjustment and efficient atraumatic suction. Taking advantage of the stability of a mechanical knob, we can obtain the relationship between the suction tube tip negative pressure and the suction tube diameter, the center negative pressure, and the air inlet size. Obviously, the tip negative pressure and central negative pressure are positive, even almost a linear correlation. Interestingly, the diameter has little effect on the tip negative pressure. And, the tip negative pressure is negatively correlated with the air inlet size, which is not obvious when the air inlet is sufficiently large. Unfortunately, we were unable to calculate a specific functional model. However, these results have guiding implications for neurosurgery. First, increasing or reducing central negative pressure is the most effective way to adjust the suction capacity of the suction tube. Second, suction tubes with very small diameters do not affect the tube tip negative pressure, which is still satisfactory for liquid transport, provided the active tip area is sufficiently large. Third, when the air inlet size is sufficiently large, there is a need to reduce the tube tip negative pressure to adjust central negative pressure.

### Recommendations of the suction tube in neurosurgery

A neurosurgical operation with the characteristics of a small and deep surgical field can be performed by one surgeon with the help of a microscope and a brain self-retaining retractor. Therefore, the suction tube should have more functions in addition to suctioning and removing blood stains and liquids. The suction tube can used to directly suction out and remove the tumor by maintaining a high tip negative pressure and pulling the normal structure under safe negative pressure, acting as a scalpel, a brain spatula, and/or a detacher. Some recommendations are provided here. First of all, “Pen-holding style” (Figure 1B) should be recommended, as it is a very solid and flexible style that helps surgeons to have access to adjust the working length and the angle of the APS tube according to the depth of the surgical field and find an appropriate fulcrum near the incision. Therefore, the length of the suction tube no longer affects the operational stability and we do not need to replace the suction tubes with a suitable length. Second, we should choose the ideal suction tube diameter and tip negative pressure. A 3.5-mm-diameter APS tube was recommended for the epidural, including the scalp and skull. Tubes with large diameter can meet the needs of removing a lot of subcutaneous bleeding from the incision of skin and the residue produced by milling a skull flap. In deep brain surgeries such as CPA, cavernous sinus, or brainstem, small-diameter APS tubes (1.5 and 2.0 mm) were recommended to reduce visual field occlusion and help protect the complex and slender neurovascular system in these areas. Unless a tip of the APS tube is inside the lesion, the air inlet (0.7–2.1 mm or one to three screw threads) should be opened to maintain a safe tip negative pressure (7.4–27.8 kPa). Third, we should continuously move the suction tube to increase the active tip area, which helps to increase suction capacity and efficiency. Finally, when it comes to operating directly on vessels, such as STA-MCA bypass, the suction tube with a diameter to match the blood vessel is recommended.

Because the existence of a load makes central negative pressure to change constantly, an accurate value of intraoperative tip negative pressure cannot be obtained but only its range, which is a major limitation of this study.

## Conclusions

Adjustable pressure suction tubes with a mechanical knob provide stable and precise tip negative pressure adjustment to prevent excessive negative pressure from causing serious intracranial structural damage. Furthermore, it allows the surgeon to hold the suction tube more freely and operate at any angle with an appropriate fulcrum near the incision to achieve efficient atraumatic suction and enhance surgical safety. In addition, this study provides some recommendations for the use of APS tubes, which are expected to be helpful to neurosurgeons in general and especially to younger neurosurgeons.

## Data availability statement

The original contributions presented in the study are included in the article/Supplementary material, further inquiries can be directed to the corresponding author.

## Ethics statement

The animal study was reviewed and approved by Experimental Animal Welfare Ethics Committee, Zhongnan Hospital of Wuhan University.

## Author contributions

PN, CX, and JC contributed to conception and design of the study. PN, MC, JibZ, MP, XG, ZL, JiaZ, WZ, XL, and JieZ contributed to acquisition and analysis of the data. PN, MC, and JC contributed to drafting of the manuscript. All authors read and approved the final manuscript.

## Funding

This study was supported by the Hubei Technological Innovation Special Fund [No. 2018ACA139].

## Conflict of interest

The authors declare that the research was conducted in the absence of any commercial or financial relationships that could be construed as a potential conflict of interest.

## Publisher's note

All claims expressed in this article are solely those of the authors and do not necessarily represent those of their affiliated organizations, or those of the publisher, the editors and the reviewers. Any product that may be evaluated in this article, or claim that may be made by its manufacturer, is not guaranteed or endorsed by the publisher.

## Supplementary material

Supplementary Video 1 This video shows the removal of a lesion adjacent to the brain stem. The adjustable pressure suction tube (APS tube) can be used to suction out and removal of the tumor without damaging the brain stem by maintaining the ideal negative tip pressure.
